# 2015 Health Survey of São Paulo with Focus in Nutrition: Rationale, Design, and Procedures

**DOI:** 10.3390/nu10020169

**Published:** 2018-02-01

**Authors:** Regina Mara Fisberg, Cristiane Hermes Sales, Mariane de Mello Fontanelli, Jaqueline Lopes Pereira, Maria Cecília Goi Porto Alves, Maria Mercedes Loureiro Escuder, Chester Luís Galvão César, Moisés Goldbaum

**Affiliations:** 1 Departamento de Nutrição, Faculdade de Saúde Pública, Universidade de São Paulo, São Paulo, SP 01246-904, Brazil; cristianehermes@yahoo.com.br (C.H.S.); marianefontanelli@gmail.com (M.d.M.F.); jaque.lps@gmail.com (J.L.P.); 2 Instituto de Saúde, Departamento de Saúde do Estado de São Paulo, São Paulo, SP 01314-000, Brazil; cecilia@isaude.sp.gov.br (M.C.G.P.A.); mmescuder@gmail.com (M.M.L.E.); 3 Departamento de Epidemiologia, Faculdade de Saúde Pública, Universidade de São Paulo, São Paulo, SP 01246-904, Brazil; clcesar@usp.br; 4 Departamento de Medicina Preventiva, Faculdade de Medicina, Universidade de São Paulo, São Paulo, SP 01246-903, Brazil; mgoldbau@usp.br

**Keywords:** Brazil, cardiometabolic risk factors, cross-sectional studies, nutrition surveys, nutrition assessment, epidemiology, biomarkers, life style, population health

## Abstract

This paper describes the design, sampling methods, and data collection procedures, with particular focus on dietary data, used for the 2015 Health Survey of São Paulo (*Inquérito de Saúde de São Paulo*, 2015 ISA-Capital) with Focus in Nutrition Study (2015 ISA-Nutrition). The ISA is a household cross-sectional, population-based survey that uses complex, stratified, multistage sampling to create a representative sample of residents from urban São Paulo, Brazil. The 2015 ISA-Nutrition comprised a sub-sample of the 2015 ISA-Capital and intended to include 300 adolescents (aged 12 to 19 years), 300 adults (aged 20 to 59 years), and 300 older adults (aged ≥60 years). From February 2015 to February 2016, 1737 individuals answered the first 24-h dietary recall (24HR), and 901 individuals consented to have their blood sample collected, to undergo anthropometric and blood pressure assessment, and to answer the second 24HR. The 2015 ISA-Nutrition aims to evaluate lifestyle-related modifiable factors in São Paulo’s residents, as well as their association with biochemical and genetic markers, and environmental aspects related to cardiometabolic risk factors. This paper concludes that 2015 ISA-Nutrition may provide valuable insights into the cardiometabolic risk factors in a big city in an upper middle-income country and contribute to the formulation of health and nutritional policies.

## 1. Introduction

In the past few decades, the surveillance and monitoring of noncommunicable diseases, such as cardiovascular diseases, and their risk factors have become a priority, due to their association with premature death, the loss of quality of life, high levels of disability, as well as economic and social effects, worldwide [1,2]. However, the multifactorial etiology of noncommunicable disease limits the efficacy of surveillance and monitoring approaches. The risk factors for cardiometabolic disease, for example, include behavioral and environmental factors, such as tobacco use, unhealthy diet, alcohol overuse, and inadequate physical activity, and physiological factors, including hypertension, and high blood cholesterol and blood glucose levels. Despite not being quantified as yet, these factors are also linked to underlying social determinants and drivers, such as globalization, urbanization, aging, income, education level, and housing type [3,4].

The 2015 Health Survey of São Paulo (2015 ISA-Capital) is a cross-sectional population-based survey with a probabilistic sample of individuals aged 12 years and older living in the city of São Paulo (Brazil), which is conducted every five years, and aims to evaluate health status, lifestyle, and use of health services [5]. A sub-sample of the 2015 ISA-Capital was drawn to compose the “2015 Health Survey of São Paulo with Focus in Nutrition Study” (2015 ISA-Nutrition).

The 2015 ISA-Nutrition aims to evaluate the association of lifestyle-related modifiable factors with biochemical and genetic markers, and the environmental factors related to the development of cardiometabolic disease, in residents of the city of São Paulo.

The study proposes a broad and profound approach to the complex relationships pertaining to noncommunicable diseases in the population, one which considers the cardiometabolic risk markers, risk behaviors, and also determinants of these behaviors, to provide useful insights for the promotion of effective public health interventions [6,7,8].

Herein, we describe the design, sampling methods, and data collection procedures, with particular focus on dietary data, involved in the 2015 ISA-Nutrition.

## 2. Experimental Section

This survey was approved by the Ethics Committee on Research of the Public Health School, University of São Paulo (certificates of presentation for ethical appreciation # 32344014.3.3001.0086 for the 2015 ISA-Capital and # 30848914.7.0000.5421 for the 2015 ISA-Nutrition). Written informed consent was obtained from all the participants and, in the case of adolescents, from their proxies too.

### 2.1. Study Population and Sampling Design

The 2015 ISA-Nutrition was a household cross-sectional, population-based survey, conducted from February 2015 to February 2016, that used stratified, multistage sampling to create a representative sample of permanent residents from the urban area of São Paulo, the most populous city in the Southern Hemisphere, located in southeastern Brazil.

To obtain the sample for the 2015 ISA-Capital, São Paulo was stratified into five sets of census tracts based on the municipality’s geographical areas for health assistance: North, Midwest, Southeast, South, and East.

In the first stage of sampling, 30 urban census tracts were randomly selected from each geographical area for health assistance, totaling 150 primary sampling units in the municipality. In the second stage, an average of 18 private households were systematically selected in each census tract. The number ‘18’ corresponded to the highest value of the households calculated, considering each demographic domain used to plan the sample: geographical area for health assistance, district/sector, age group, and sex. All individuals in the households who belonged to the demographic domain selected in the study were invited.

The goal was to obtain a final sample of 4250 individuals (808 adolescents (age 12 to 19 years), 2462 adults (age 20 to 59 years), and 980 older adults (age ≥60 years)), with 850 individuals in each geographical area for health assistance.

To minimize the effects of losses and refusals to participate in the following phases, a higher number of independent random selections were made. Different sampling fractions were used to select the individuals belonging to the domains. To deal with these differences, sampling weights are used in the data analysis. Further details on the 2015 ISA-Capital can be found elsewhere [9].

The 2015 ISA-Nutrition was composed of a sub-sample of the 2015 ISA-Capital, as part of which the plan was to include 300 adolescents (age 12 to 19 years), 300 adults (age 20 to 59 years), and 300 older adults (age ≥60 years). The number ‘300’, by domain, allows for the estimation of the proportions of the changes/differences of 0.50, with a sampling error of seven percentage points, considering a 95% confidence level of the population value being within the lower and upper limits, and a design effect of 1.5.

**Figure 1:**
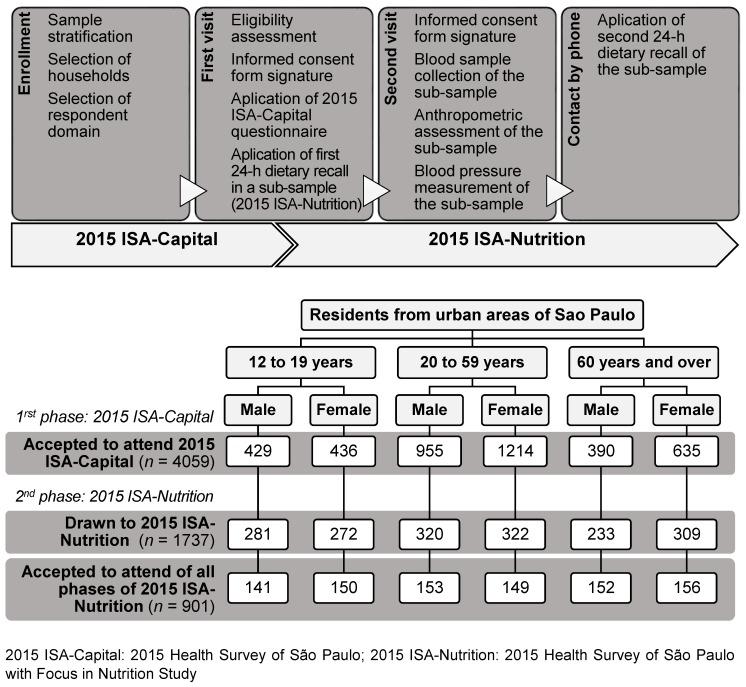
Study design and description of the sample number in the 2015 Health Survey of São Paulo with Focus in Nutrition Study (2015 ISA-Nutrition). São Paulo, 2015.

Of those who agreed to participate in the 2015 ISA-Capital (*n* = 4059), 1737 individuals were randomly selected to the 2015 ISA-Nutrition, and they answered the first 24-h dietary recall (24HR) (Figure 1). These participants were invited to take part in the second phase of the survey, and 901 individuals agreed to have their blood sample collected, undergo anthropometric and blood pressure assessments, and answer the second 24HR.

Individuals with chronic alcoholism, those on an enteral and/or a parenteral diet, and pregnant/lactating women were excluded from the 2015 ISA-Nutrition. The response rate to the 2015 ISA-Nutrition (second phase) was 52%. In the second phase of the 2015 ISA-Nutrition, 21% of the participants immediately refused to participate, 10% initially agreed to participate and, afterwards, declined, 13% could not be contacted (invalid telephone number, did not answer the phone, or the person who answered did not know the participant), 2.5% had changed their addresses, 1.2% were excluded, and 0.2% died between the first and second phases.

### 2.2. Study Design

**Figure 2:**
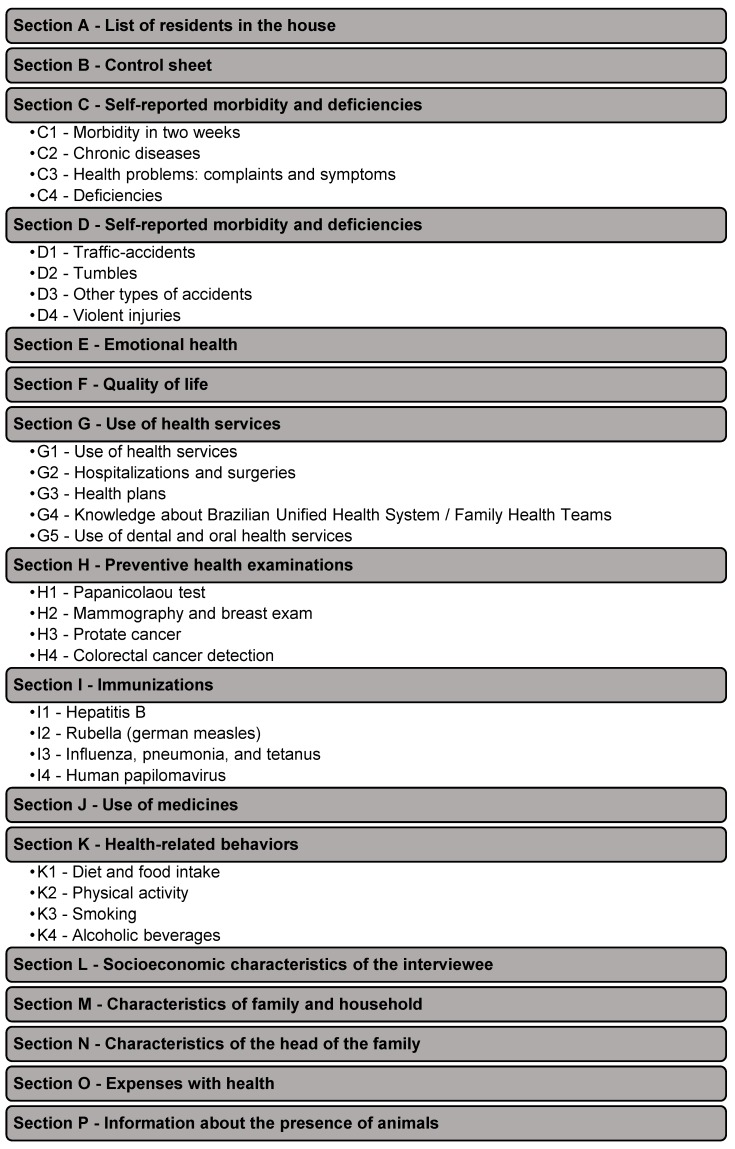
Sections of the questionnaire used in the 2015 ISA-Capital. São Paulo, 2015.

After the households were selected, trained interviewers visited the selected households to conduct face-to-face interviews based on a structured questionnaire (the 2015 ISA-Capital questionnaire) [4], which was formulated so as to be applied using a tablet device (Figure 1). The sections of the questionnaire are shown in Figure 2.

During the first visit, a sub-sample of individuals who were previously randomly selected were invited to answer the first 24HR. The respondents were invited to the second phase and received an informative flyer on the procedures to be undertaken in the second household visit. For those who agreed to continue, the second visit was scheduled by phone, and the procedures for blood collection were explained: 12-h fasting and no alcoholic beverage consumption for 72 h preceding the collection, and no intense physical activity prior to and on the day of blood collection.

The second visit occurred approximately 48 days after the first visit, and it was conducted by trained nurses who performed venous blood sample collection (~30 mL) using Vacutainer tubes, anthropometric assessment (weight, height, waist circumference), and blood pressure measurement. In this phase, another informed consent form was signed, and the use, if any, of drugs and/or supplements, was recorded.

**Figure 3:**
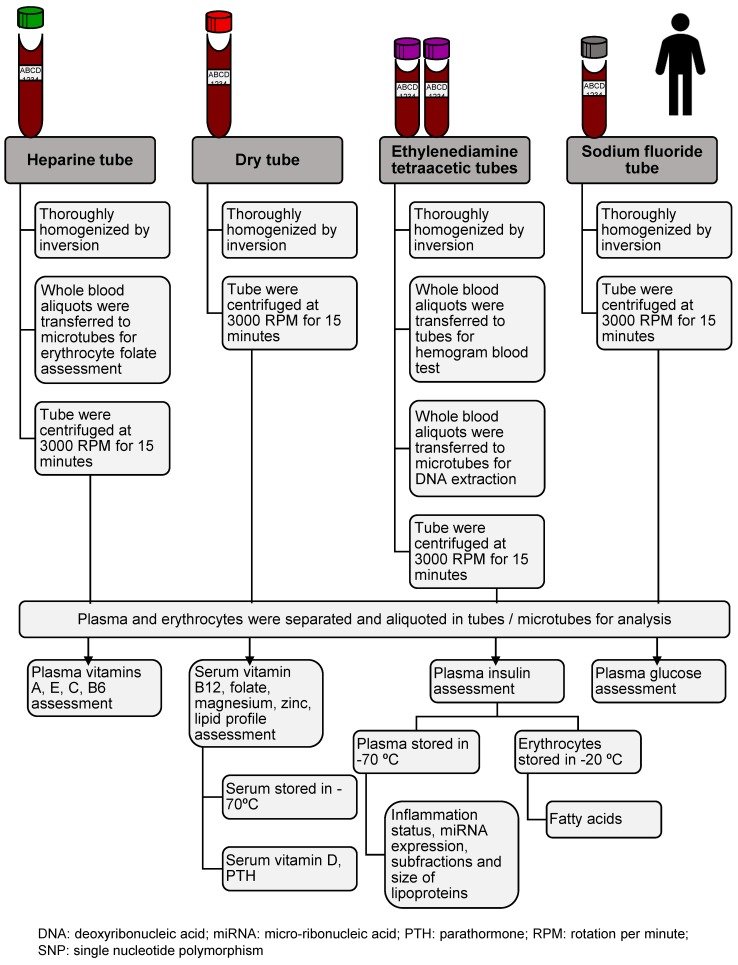
Laboratory procedures used for blood sample assessment: 2015 ISA-Nutrition. São Paulo, 2015.

The blood samples were collected after 12 to 14 h of fasting and were sent to the Laboratory of Nutritional Genomics and Inflammation of the Public Health School of the University of São Paulo, in Styrofoam boxes containing recyclable ice. Standardized specific procedures were then performed (Figure 3). Nine aliquots, per participant, were immediately forwarded to the laboratory for analysis. Approximately 25 aliquots were stored according to the requirements for each dosage at −20 °C or −70 °C. The baseline laboratory measurements used in the 2015 ISA-Nutrition are shown in Table 1.

A mean of 173 days after the second visit, trained nutrition students contacted the participants via telephone to perform the second 24HR and confirm if they had received the results of their biochemical analyses at their home or via e-mail.

### 2.3. Emotional Health

Physical and psycho-emotional symptoms were determined using the Self-Reporting Questionnaire, which was validated in São Paulo [11].

### 2.4. Physical Activity Practice

Physical activity was assessed using the long International Physical Activity Questionnaire, which was validated in the Brazilian population [12,13]. Individuals were classified as “meet the recommendation” or “do not meet the recommendation” according to the latest recommendations for physical activity of the World Health Organization (WHO) [14].

### 2.5. Alcohol Consumption Habits

The consumption of alcoholic beverages was measured using the Alcohol Use Disorders Identification Test [15]. This instrument addresses alcohol dependence, harmful consumption, and hazardous alcohol consumption, and was validated in Brazil [16].

### 2.6. Dietary Assessment

Dietary intake data were collected through two dietary recall questionnaires performed on nonconsecutive days (Table 2). In this method, data of all the foods and beverages consumed by the individual during the day before the interview were used. The interviewer who applied the 2015 ISA-Capital structured questionnaire collected, in paper form, the responses to the first 24HR from the participants’ homes. The interviewers were previously trained in the Multiple Pass Method [17]. In this method, interviewers collect dietary data in five successive steps, to keep the individual interested and engaged in the interview, and to help them in remembering all the items consumed.

The second 24HR was collected via telephone 185 days (median) after the first 24HR. Interviews were conducted electronically by trained nutrition students, assisted by the Nutrition Data System for Research (NDSR) software (version 2014) developed by the Nutrition Coordinating Center, University of Minnesota, Minneapolis, MN, the United States of America [18].

In both collections, the interviewers were instructed to record the food consumption in household measures, and to ask questions about the preparation modes, ingredients, and trademarks of the food items. Household measures were converted into units of weight and volume [19,20]. This procedure was undertaken to standardize the entry of the data in the NDSR software. The nutritional values of energy and macronutrients of all the foods included in the 2015 ISA-Nutrition database were compared to those in the Brazilian food composition table, and when the values differed between the databases, they were corrected to correspond to those of the foods consumed by the population of São Paulo [21]. The nutritional values of regional foods and preparations were estimated based on the Brazilian food composition table [21].

Dietary supplement intake was investigated during the first 24HR interview, and on the day of blood collection. Individuals were requested to report the brand name or the active ingredient and the dosage of the medication they were currently taking. For supplements described only by brand name, the nutrient information was identified for further classification. 

The usual dietary intake was estimated using statistical modeling techniques incorporated into the Multiple Source Method program—an online platform that calculates the usual intake of nutrients and foods consumed by the population based on repeated measurements of the 24HR [22].

### 2.7. Anthropometric Measurements

The weight and height of participants were measured in triplicate, with the participants barefoot and wearing light clothes. The weight was measured in kilograms using a calibrated platform-type digital scale (Tanita^®^, model HD-313, Arlington Heights, IL, USA, maximum capacity of 150 kg, and precision of 100 g). A portable stadiometer (Seca^®^, model 208, Cotia, SP, Brazil, maximum measurement of 200 cm, and precision of 0.1 cm) fixed on a smooth wall, without a baseboard, was used for the measurement of height in centimeters.

Data on weight, height, and waist circumference were collected according to the WHO guidelines [23]. For the weight measurement, individuals were asked to stand at the center of the balance platform, in an upright position with parallel and joined feet, and with their arms placed along the body. For the height measurement, individuals were positioned in the Frankfort plane, with their heels, calves, buttocks and shoulders, and the backs of their heads touching the vertical surface of the stadiometer. Participants were asked to remove any adornments that could interfere with the measurement. Waist circumference was measured using inextensible metric tape, positioned above the midpoint, between the last costal arch and the iliac crest of the standing participants, during expiration.

The mean values of the weight and height of each participant were used to calculate the body mass index (BMI). The BMI values were used to classify the participants’ anthropometric nutritional statuses, according to standardized criteria for each life stage [24,25,26]. The waist circumference was used to diagnose central obesity, according to the criteria defined by the WHO [23].

### 2.8. Blood Pressure Measurement

Blood pressure was measured using an automatic device (Omron, model HEM-712C, Omron Healthcare, Inc., Kyoto, Japan). Two blood pressure measurements were taken using a cuff adequate for the brachial circumference of the participants. The measurements were taken after participants rested for 5 min in a sitting position, with their arm supported at heart level. Blood pressure was initially measured in the right arm, and, 1 min after the first measurement, it was assessed in the left arm. One additional measurement was obtained in the arm with the largest value. If there was a difference greater than 10% between the measures, a third measure was undertaken. The results of the systolic and diastolic pressures are expressed in arithmetic means, in mmHg.

### 2.9. Data management, Quality Assurance Strategies, and Dissemination Plans

The 2015 ISA-Nutrition group developed a comprehensive data capture and management system along with quality control to ensure rigorous and high-quality data collection. Standard operating procedures (SOPs) were formulated to ensure the same methods were applied to all procedures: the 2015 ISA-Capital questionnaire was administered in the households using tablet devices, dietary intakes were assessed in the households and through telephone calls, the household measures reported in the 24HR were converted into units of weight and volume, anthropometric and blood pressure assessments were performed, blood was collected, and blood samples were processed and analyzed.

Interviewers were selected by their abilities, and then trained according to the SOPs. Periodically, meetings were held by coordinating staff to check if standardized procedures were used accurately and to discuss interviewers’ doubts and problems. The collection of the 2015 ISA-Capital questionnaire was performed using tablet devices, and interviewers updated the system daily to generate a database, which was monitored by a database management system and transmitted to the coordinating staff for data checking. For quality control, coordinating staff, at random, telephoned some of the participants and asked them some questions to confirm the values obtained by the interviewers. This process verified the inexistence of fraudulent entries in the data. At every staff meeting, 24HR data were delivered, and checked by a trained nutritionist, so that feedback on the quality of the dietary assessment could be provided to each interviewer.

Procedures to ensure consistency of the procedures were followed to detect possible typing errors in all the datasets, using an independent double-checking process. In the dietary dataset, the 24HR data of individuals with energy intakes below 800 kilocalories per day or above 4000 kilocalories per day were reviewed for possible errors. Micronutrient intake was compared to those in the Brazilian food composition table, and values outside 80% and 120% of concordance were corrected using a data correction routine elaborated for the Stata software (version 13.0, StataCorp LP, College Station, TX, USA).

The final dataset is held by the coordinating staff in different storage media: magnetic, optic, and electronic, and in the cloud. Investigators who intend to analyze the 2015 ISA-Capital data must obtain study approval from the coordinating staff. Once approved, the dataset will be provided to the investigator, who will be instructed to consider the complexity of the sample. A multifaceted approach will be used to disseminate the study’s findings, such as through professional and community meetings, publications in peer-reviewed journals, and press releases.

## 3. Discussion

The 2015 ISA-Nutrition was conducted in São Paulo, which is a destination for many national and international migrants [27]. In this scenario, the 2015 ISA-Nutrition may provide valuable general insights into the risk factors for cardiometabolic disease in a big city of a middle-income country.

Nutrition surveys, such as the Japan National Health and Nutrition Survey (the oldest national health survey in the world) [28], the United States’ National Health and Nutrition Examination Survey [29], and the National Diet and Nutrition Survey [30] from the United Kingdom are a few examples of investigations that have been providing evidence on the diet and nutrition of large populations, thus enabling the monitoring of public health nutrition objectives. In Brazil, the Brazilian Household Budget Survey (POF) [31], the Risk and Protective Factors Surveillance System for Chronic Diseases by Telephone Interviews (Vigitel) [32], the National Health Survey (PNS) [33], and the National Sample Survey of Households (PNAD) [34] have contributed to the current needs of the Brazilian Health System (SUS). It is worth noting that, even at a municipal level, 2015 ISA-Nutrition was the first health survey to collect biological material in a population-based sample, in Brazil. Nowadays, although other national initiatives have emerged, our study highlights the wide range of biochemical measurements and the complexity of cardiometabolic risk factors involved in the 2015 ISA-Nutrition; these factors still have gaps in the literature, especially in the context of a big city of a middle-income country.

Regarding dietary assessment, it is important to mention that the major source of food data of the NDSR is the United States Department of Agriculture (USDA)’s food composition table, and the nutritional composition of the foods consumed by the population of São Paulo was obtained from these tables. Therefore, nutrient-related information was compared and corrected according to the Brazilian food database [21]. However, it is important to recognize that the Brazilian tables are also limited, in terms of the foods and nutrients included, thereby leading to the use of international food composition tables and the nutritional information provided by the industry, when necessary. In relation to fortified foods, interviewers were instructed to record the trademarks of the items consumed. When this information was available, the specific nutrient profile of the food consumed was taken into account, but the names of the food brands were not available for all the foods consumed; this constitutes a limitation of our study. However, the fortification of foods with iron and folic acid—mandatory in Brazil since 2004—was considered by statistical routines.

The time difference between the performance of the dietary assessment methods may be another limitation; however, there is no consensus on the optimal interval between the first and second 24HR for the estimation of usual dietary intake. Although one 24HR is enough to accurately estimate the mean intake of the population, the application of the second 24HR to at least a sub-sample is important to estimate the usual dietary intake, which is necessary to estimate the distribution of intakes in the population, investigate the association with blood nutrient status, and examine the association with diet as an independent or dependent variable [35]. The 24HR questionnaires in the 2015 ISA-Nutrition were administered on non-consecutive days, so as to represent all the days of the week and seasons of the year, using a standardized interviewing process to ensure consistency among interviewers. Another source of bias in the dietary assessment may be the methods used for dietary collection. The first 24HR was collected via face-to-face interviews, and, the second, by telephone calls. Data from the 2008 ISA-Nutrition suggest that the rates of underreporting were higher in the telephone interviews than face-to-face interviews (the median underreporting percentage of energy intake was 38.9% of the energy needs in the face-to-face interview, and 40.2% in the telephone interview (*p* = 0.013)) [36]. It is worth mentioning that conducting two face-to-face interviews would increase the cost of this population-based survey.

São Paulo’s population is ethnically and culturally very diverse; this is reflected in its dietary intake. The foods and recipes reported in the dietary recalls of the 2015 ISA-Nutrition varied from the typical regional foods of Brazil to those from other countries; this made the food standardization procedure a challenging task. 

In addition, there are sociodemographic and epidemiological differences across the areas in the city, which were accounted for in the sampling design. For example, the population is older in the Midwest and Southeast regions, while the mortality coefficients for selected causes (cervical cancer, ischemic heart disease, cerebrovascular disease, and diabetes mellitus) and the years of life lost due to premature mortality are both the highest in the East and the lowest in the Midwest region [5]. These differences reflect the diversity in terms of healthcare needs and may also account for the differences in the participation rates in the surveys.

Another limitation of the study is the high nonresponse rate. Usually, in household surveys, there is a need to consider the nonresponse of households and individuals: the first refers to the refusal of the residents to open up their homes and/or to inform the existence of any individual belonging to the study population, and the second refers to the impossibility of conducting interviews with residents identified as belonging to the sample. In the 2015 ISA-Capital, the response rates were 76% for the household and 73% for the individual. However, for the 2015 ISA-Nutrition, another research step should be considered—the realization of clinical examinations, which could give rise to another possibility of nonresponse. The refusal to provide material for clinical examinations (31%), and the difficulty in finding individuals in the same address in which they were previously interviewed in the 2015 ISA-Capital (16%) should be considered in this step. Together, these two percentages correspond to almost half of the individuals who should be examined. It is important to disseminate this outcome so that the researchers involved in the epidemiologic surveys are aware of the challenges to be faced. There are currently few data in the literature which focus on nonresponse in this type of study [9].

Despite the limitations, the notable strengths of the 2015 ISA-Nutrition include its multiple-edition character, allowing for the monitoring of dietary habits and cardiometabolic risk factors in São Paulo’s population, over a period of more than ten years (2003–2015). The first edition of the ISA-Nutrition was conducted in 2003 with only one 24HR, in a large sample of the city’s residents (2376 participants). In 2008, the survey had two 24HR measures in a sub-sample of the 2008 ISA-Capital (1662 individuals), and a second home visit was made to obtain anthropometric and blood pressure measurements, and to collect blood samples. The consistency in the methods used in the surveys over the years allows for several comparisons to be made in terms of the health and diet issues in the population across the entire time period.

In addition, the population-based sampling procedure used is also an advantage of this survey, as it captures the social and ethnic diversity of São Paulo. The regularity of health surveys can provide insights on evolutionary trends, and support managers in decision making [37]. The ISA-Capital is conducted an average of once every five years, in partnership with the University of São Paulo and the São Paulo Municipal Health Department. This is the third version of ISA-Nutrition. The earlier editions were those from which data on diet were collected.

## 4. Conclusions

The findings of the multiple studies performed for this survey may contribute to gaining a better understanding of the gaps in the literature regarding the traditional cardiometabolic risk factors, and for establishing a better understanding of the emerging risk factors for the residents of São Paulo. Therefore, our study’s findings may assist in the monitoring and planning of health policies and programs.

## Figures and Tables

**Table 1:** Baseline laboratory measurements in the 2015 ISA-Capital. São Paulo, 2015.

Analyte	Sample	Technical Reference
Vitamin A (retinol)	Heparinized plasma	High performance liquid chromatography method (Ref. # KC 1600; Immundiagnostik AG, Bensheim, HE, Germany)
Vitamin C (ascorbic acid)	Heparinized plasma	High performance liquid chromatography method (Ref. # KC 2900; Immundiagnostik AG, Bensheim, HE, Germany)
Vitamin E (alpha-tocopherol)	Heparinized plasma	High performance liquid chromatography method (Ref. # KC 1600; Immundiagnostik AG, Bensheim, HE, Germany)
Vitamin D (cholecalciferol-25 (OH)D3)	Serum	Chemiluminescence (Diasorin kit, Stillwater, MN, USA)
Vitamin B6 (pyridoxal-5-phosphate)	Heparinized plasma	High performance liquid chromatography method (Ref. # KC 2100; Immundiagnostik AG, Bensheim, HE, Germany)
Vitamin B9 (folate)	Serum and erythrocytes	Chemiluminescent immunoassay (Ref. # A98032; Beckman Coulter Inc.; Fullerton, CA, USA)
Vitamin B12 (cobalamin)	Serum	Chemiluminescent immunoassay (Ref. # 3300; Beckman Coulter Inc.; Fullerton, CA, USA)
Parathormone	Serum	Elecsys 2010 kit, Roche Diagnostics, Indianapolis, ID, USA
Iron	Serum	Colorimetric assay (Cobas; Roche Diagnostics GmbH, Mannheim, BW, Germany)
Ferritin	Serum	Chemiluminescent immunoassay (Ref. # 33020; Beckman Coulter Inc.; Fullerton, CA, USA)
Transferrin	Serum	Kinetic nephelometry (Ref. # OSR6152; Beckman Coulter Inc.; Fullerton, CA, USA)
Whole blood cell count	Whole blood	Automatic method
Magnesium	Serum	Photometric color assay (Ref. # OSR6189; Beckman Coulter Inc.; Fullerton, CA, USA)
Zinc	Serum	Flame atomic absorption method (AAnalyst 100 instrument; Perkin Elmer, Norwalk, CT, USA) [10]
Total cholesterol	Serum	Trinder reaction (cholesterol oxidase) (Cobas; Roche Diagnostics GmbH, Mannheim, BW, Germany)
Low-density lipoprotein-cholesterol	Serum	Homogeneous enzymatic colorimetric assay (Cobas; Roche Diagnostics GmbH, Mannheim, BW, Germany)
High-density lipoprotein-cholesterol	Serum	Homogeneous enzymatic colorimetric assay (Cobas; Roche Diagnostics GmbH, Mannheim, BW, Germany)
Triacylglycerol	Serum	Enzymatic colorimetric assay (glycerol phosphate peroxidase) (Cobas; Roche Diagnostics GmbH, Mannheim, BW, Germany)
Very low-density lipoprotein-cholesterol	Serum	Values were calculated by dividing the triglyceride values by five
Non-high-density lipoprotein-cholesterol	Serum	Values were determined as the difference between the values of total cholesterol and high-density lipoprotein-cholesterol
Glucose	Sodium fluoride plasma	Colorimetric enzymatic assay of glucose oxidase (Trinder reaction) (Cobas; Roche Diagnostics GmbH, Mannheim, BW, Germany)
Insulin	Ethylenediamine tetraacetic plasma	Multiplex immunoassay (LINCOplex^®^; Linco Research Inc., St. Charles, MO, USA)

**Table 2:** Characteristics of the individuals who answered the 24-h dietary recall (24HR) questionnaires. São Paulo, 2015.

Variables	First 24HR (In-Person)	Second 24HR (Phone)	Both 24HR
Total (*n*)	1744	548	2292
Age category (%) ^a^			
Adolescent (12 to 19 years)	31.8	33.0	32.1
Adult (20 to 59 years)	36.9	28.8	35.0
Older adult (≥60 years)	31.3	38.1	32.9
São Paulo geographical area for health assistance (%)			
North	14.8	13.5	14.5
Midwest	17.6	20.4	18.3
Southeast	22.5	23.4	22.7
South	26.3	25.2	26.0
East	18.9	17.5	18.6
Weekend; Saturday, Sunday (%)	31.1	19.3	28.3
Intake reliability (% Reliable) ^b^	94.0	91.4	93.4
Intake amount (%)			
Close to the amount usually eaten	84.8	75.2	82.5
A lot more than usually eaten	5.8	8.2	6.4
A lot less than usually eaten	9.4	16.6	11.1
Energy intake (%)			
Less than 800 kcal	5.4	6.0	5.6
More than 4000 kcal	2.8	2.0	2.6

^a^ Mean age according to age category: adolescent = 15.5 y, adult = 39.3 y, older adult = 69.8 y. ^b^ Interviewer’s answer to the question: “Do you consider that the information collected from the interviewee was…” “Reliable”, “Unreliable because the participant was unable to recall one or more meals” or “Unreliable for other reasons (cite)”, after the 24HR collection. 24HR: 24-h dietary recall.
